# Bacterial contamination of vegetables sold in Arba Minch Town, Southern Ethiopia

**DOI:** 10.1186/s13104-018-3889-1

**Published:** 2018-10-30

**Authors:** Getaneh Alemu, Mohammedaman Mama, Munira Siraj

**Affiliations:** 1 Department of Medical Laboratory Science, Bahir Dar University, Bahir Dar, Ethiopia; 2 Department of Medical Laboratory Science, Madda Walabu University Goba Referral Hospital, Bale-Robe, Ethiopia; 3 Department of Medical Laboratory Science, Arba Minch University, Arba Minch, Ethiopia

**Keywords:** Vegetables, Contamination, Bacteria

## Abstract

**Objective:**

Unhygienically handled fruits and vegetables which are usually consumed in raw serve to transmit various infectious diseases. Bacteria are among the common vegetable contaminants. However, the species of contaminants and rate of contamination depends on various environmental and human factors. Hence, a cross-sectional study was conducted to assess the level of bacterial contamination and associated factors among vegetables marketed in Arba Minch town from January to March, 2018. A structured questionnaire was used to collect data regarding factors associated with bacterial contamination of vegetables. Selected vegetables were purchased and processed for examination of bacterial contamination by standard culture technique following standard protocols. All data were analyzed using SPSS version 20.0.

**Results:**

A total of 347 vegetable samples were examined, of which 169 (48.7%) were positive for bacteria contamination. Cabbage (71.9%) was the most frequently contaminated vegetable. *E. coli* (31.4%) was the most frequent contaminant detected. Type of vegetables (p = 0.000) and market place (p = 0.039) show significant association with bacterial contamination. Bacterial contamination rate in the present study was significantly considerable. Therefore we recommend for the local health office to continuously monitor the contamination status of raw edible vegetables and take respective measures.

## Introduction

Fruits and vegetables are highly beneficial components of balanced diet which play pivotal role for maintenance of health and prevention of disease [1]. They are rich in carbohydrate, vitamins, minerals and fiber contents [2–4]. As a result, the World Health Organization (WHO) recommended the intake of a minimum of 400 g of fruits and vegetables per day not only for the prevention of chronic diseases like heart disease, cancer, diabetes and obesity; but also for the prevention and alleviation of several micronutrient deficiencies [4, 5]. On the contrary, unhygienically prepared and consumed fruits and vegetables could bring potential risk of acquiring various infectious diseases. The risk of disease transmission is much higher among fruits and vegetables consumed in raw and/or unwashed [2, 3, 6, 7].

Enteric bacterial infections are distributed virtually throughout the world and have been causing morbidity most commonly associated with infections in communities where poor environmental sanitation and personal hygiene are prevalent [8]. As much as 70% of diarrheal diseases in developing countries are believed to be of food borne origin [9, 10]. Vegetables can be contaminated with enteric bacteria of medical and public health importance during cultivation, harvest, transportation and further processing. As a result, they have been mentioned in many of previous food-borne outbreaks [11]. The poor personal and environmental hygiene and poor health system commonly observed in developing countries make the prevalence to be highest among the population in those countries [1, 5].

Enteric bacteria notably *Salmonella, Escherichia* and *shigella species* continue to be major global health problems and are the leading causes of morbidity and mortality in both developed and developing countries [9]. The increase in these food-borne infections may have resulted from increased consumption of contaminated fruits and vegetables [12].

Several factors may contribute to contamination of vegetables. Vegetables may get exposed to bacterial contaminants in the pre-harvest and post-harvest handling [13, 14]. Use of insufficiently treated wastewater for irrigation is an important risk factor for contamination of vegetables cultivated using irrigation in many of developing countries [13]. Contamination of soil with animal wastes and increased application of improperly composted manures to soil has also similar role [15]. Poor or inappropriate hygienic practice during production, transport, processing and preparation by handlers including consumers also contribute in vegetable contaminations [16].

If our target is to control infectious diseases, it is not enough to depend merely on chemotherapeutic intervention, but need the concerted effort to reduce and eliminate the potential sources of infection. To cope up with this effort, periodic detection of medically important infectious contaminants of vegetables and associated practices seek priority attention [17]. Therefore, the aim of the present study was to determine the level of bacterial contamination of selected vegetables and associated factors in Arba Minch Town, Ethiopia.

## Main text

### Methods and materials

#### Study design and area

A cross-sectional study was conducted from January to March 2018 in local markets of Arba Minch town, Southern Ethiopia. The town is located 454 kms away in the southern direction from Addis Ababa, capital city of Ethiopia. It is found at an altitude of 1200–1300 meters above sea level with an average annual temperature of 29.7 °C and rain fall of 900 mm [11]. The weather condition during our data collection period was hot and dry during January and February, and the minor rainy season starts in March. The climate of the town and surrounding rural area is conducive for cultivation of different fruits and vegetables. As a result, diverse types of fruits and vegetables are frequently utilized as sources of food in the town. Those fruits and vegetables are readily available in shops, super markets and open markets throughout the town and most types are eaten in raw.

#### Sample collection

A structured questionnaire administered to local vendors by face to face interview was used to collect data about factors associated with parasitic contamination of vegetables. About 200 gm of vegetables were purchased from four local markets (Sikela, shecha, Yet nebersh and Konso sefer). Variable numbers of samples were collected from each vegetable type based on their abundance in markets. Accordingly, 100 *Lycopersicon esculentum* (tomato), 96 *Brassica oleracea* (cabbage), 66 *Capsicum annuum* (Green pepper), 62 *Daucus carota* (carrot) and 23 *Lactuca sativa* (lettuce) (totally 347) samples were collected. Samples were collected, put in sterile plastic bags, labeled, and transported through transporting cold box to the Medical Microbiology and Parasitology Teaching Laboratory of College of Medicine and Health Sciences for parasitological analysis. The Analysis was done within 24 h after collection.

#### Isolation of bacteria

Samples were transported in sterile packet and analyzed for isolation and identification of pathogenic bacteria following standard methods. About 25 gm of vegetable samples were rinsed thoroughly with sterile water and tenfold serial dilutions of each rinse water was made and 1 ml of 10^−2^, 10^−4^, 10^−5^, 10^−6^ was pipetted into MacConkey, *Salmonella Shigella* agar, Manitol Salt agar and nutrient agar using the Pour Plate Technique. The plates were allowed to solidify, inverted and incubated at 37 °C for 24 h for colony formation. Distinctive morphological properties of each pure culture such as colony form, elevation of colony and colony margin were observed. Bacterial pathogens were isolated and identified on the basis of morphological, cultural and different biochemical tests following standard protocol as described elsewhere [18].

### Statistical analysis

Both questionnaire and laboratory data were cleaned entered and analysed using SPSS version 20.0. The proportion of vegetables contaminated with different types of medically important bacteria were computed. Chi square test and logistic regression analysis were used to identify factors associated with bacteria contamination. Association between variables was considered statistically significant only if p-value ≤0.05 at 95% confidence level. The strength of association was measured through adjusted odds ratios.

### Results

A total of 347 vegetable samples were collected from local markets and examined for bacterial contamination. Vegetables were collected from four markets namely Sikela (230, 66.3%), Shecha (57, 16.4%), Konso sefer (36, 10.4%) and Yetnebersh (24, 6.9%). Vegetables collected were Tomato (100, 28.8%), Cabbage (96, 27.7%), Green pepper (66, 19.0%), Carrot (62, 17.9%) and Lettuce (23, 6.6%). Results of this study showed that 169 (48.7%) vegetable samples were contaminated with bacteria. Cabbage was the most frequently contaminated vegetable (69, 71.9%) followed by carrot (35, 56.5%). Four species of bacteria were detected *E. coli* being the commonest bacterial contaminant of vegetables (109, 31.4%) (Table 1).

**Table 1:** Frequency distribution of bacterial contamination among vegetables sold in local markets of Arba Minch Town from January to March 2018

Vegetable type	Number examined	Number contaminated with each species of bacteria				Total contaminated (%)
		*E. coli*	*S. aureus*	*Shigella* spp.	*Salmonella* spp.	
Tomato	100	20	4	0	9	29 (29.0)
Cabbage	96	44	31	21	23	69 (71.9)
G. pepper*	66	14	4	7	8	26 (39.4)
Carrot	62	26	7	18	3	35 (56.5)
Lettuce	23	5	0	3	3	10 (43.5)
Total (%)	347	109 (31.4)	46 (13.3)	49 (14.1)	46 (13.3)	169 (48.7)

The Chi square (Χ^2^) test result show difference in bacterial contamination rate among different kinds of vegetables (p = 0.000). Further binary logistic regression analysis showed that tomato was 2.244 (AOR: 2.244; 95% CI 1.112, 4.529) times more likely to be contaminated with bacteria as compared to green pepper. On the contrary cabbage was 73.5% less likely to be contaminated (AOR: 0.265; 95% CI 0.133, 0.529). Market place was also significantly associated with bacterial contamination (p = 0.039). Vegetables from Sikela (AOR: 0.436; 95% CI 0.192, 0.991) and Shecha (AOR: 0.143; 95% CI 0.052, 0.394) were significantly protected from bacterial contamination compared to vegetable samples collected from Konso sefer (Tables 2 and 3).

**Table 2:** Chi square test of factors associated with bacterial contamination of vegetables sold in local markets of Arba Minch Town from January to March 2018

Variables	Category	Number examined	Rate of parasite contaminated N (%)	Χ^2^	p-value
Vegetable type	Tomato	100	29 (29.0)		
	Lettuce	23	10 (43.5)		
	Cabbage	96	69 (71.9)	40.107	0.000
	Carrot	62	35 (56.5)		
	Green pepper	66	26 (39.4)		
Washed before display	Yes	49	27 (55.1)	0.958	0.334
	No	298	142 (47.7)		
Market	Sikela	230	109 (47.4)		
	Yetnebersh	24	12 (50.0)	8.346	0.039
	Shecha	57	36 (63.2)		
	Konso sefer	36	12 (33.3)		
Means of display	On the floor	338	166 (49.1)	0.874	0.350
	On table/shelf	9	3 (33.3)		
Handled by vendor who has	No formal edu	36	15 (41.7)	1.196	0.550
	Primary school	290	145 (50.0)		
	Secondary/above	21	9 (42.9)		
Source of vegetables	Farmers	260	120 (46.2)	2.986	0.100
	Large scale vendors	87	49 (56.3)

**Table 3:** Binary logistic regression of factors associated with bacterial contamination of vegetables sold in local markets of Arba Minch Town from January to March 2018

Variables	Category	Number examined	Rate of bacteria contam N (%)	COR (95% CI)	p-value	AOR (95% CI)	p-value
Vegetable type	Tomato	100	29 (29.0)	1.632 (0.845, 3.151)	0.144	2.244 (1.112, 4.529)	0.024
	Lettuce	23	10 (43.5)	0.933 (0.361, 2.416)	0.887	0.981 (0.369, 2.605)	0.969
	Cabbage	96	69 (71.9)	0.261 (0.134, 0.508)	0.000	0.265 (0.133, 0.529)	0.000
	Carrot	62	35 (56.5)	0.514 (0.254, 1.042)	0.065	0.542 (0.262, 1.121)	0.098
	Green pepper	66	26 (39.4)	1			
Washed before display	Yes	49	27 (55.1)	1			
	No	298	142 (47.7)	1.348 (0.735, 2.474)	0.335		
Market	Sikela	230	109 (47.4)	0.555 (0.265, 1.163)	0.119	0.436 (0.192, 0.991)	0.047
	Yetnebersh	24	12 (50.0)	0.500 (0.173, 1.441)	0.199	0.574 (0.174, 1.898)	0.363
	Shecha	57	36 (63.2)	0.292 (0.121, 0.701	0.006	0.143 (0.052, 0.394)	0.000
	Konso sefer	36	12 (33.3)	1			
Means of display	On the floor	338	166 (49.1)	0.518 (0.127, 2.106)	0.358		
	On table/shelf	9	3 (33.3)	1			
Handled by vendor who has	No formal edu	36	15 (41.7)	1.050 (0.353, 3.120)	0.930		
	Primary school	290	145 (50.0)	0.750 (0.307, 1.834)	0.528		
	Secondary/above	21	9 (42.9)	1			
Source of vegetables	Farmers	260	120 (46.2)	1			
	Large scale vendors	87	49 (56.3)	0.665 (0.408, 1.084)	0.102	0.383 (0.196, 1.745)	0.105

AOR, adjusted odd ratio; CI, confidence interval; COR, crude odd ratio

### Discussion

Bacterial contamination rate of vegetables in the present study was considerable. However, it is much lower than the same study result conducted in the same area before 9 years where all the vegetable samples examined were contaminated with *E. coli* [19]. Actually only tomato and lettuce were considered in the previous study and presence of bacteria other than *E. coli* were not considered. Data was collected in the dry season that the tendency of contamination due to flood was less in the present study. Cabbage was the most frequently contaminated vegetable in the present study (71.9%). This is in contrast to previous study in Arba Minch where tomato was predominantly contaminated. It is justifiable that cabbage has large surface area and course surface which enables to attach contaminants as compared to smooth surfaced vegetables with narrow exposed outer surface like tomato.

Among the four bacterial species identified, *E. coli* was the commonest contaminant (31.4%). This goes in line with findings from Istanbul (41.7%), Brazil (40–41.5%), Vietnam (100%) and Bangladesh (36%) [20–23]. *E. coli* is a fecal coliform bacterium usually excreted in stool that it is obvious to be abundant in the environment where open defecation is common (like Ethiopia). In addition, farmers usually use human excreta as a natural fertilizer; this accelerates rate of contamination of vegetables cultivated in such farms. However, the rate of *E. coli* contamination in this study is lower as compared to previous results from Istanbul, Brazil and Vietnam [20–22]. On the other hand, studies from Nigeria and Sudan indicated *Staphylococcus species* as the most frequent bacterial isolate [12, 24]. These differences might be due to variations in the time (season) of data collection and sensitivity of laboratory methods used.

The other three bacterial species detected have almost equal level of incidence; *S. aureus* (13.3%), *Shigella species* (14.1%), and *salmonella species* (13.3%). Significantly higher contamination rate of *S. aureus* was observed in previous studies conducted in Bangladesh (24%), India (56–74%) and Nigeria (29.2%) [12, 23, 25]. *Salmonella species* were also more common contaminants than the present study result according to previous results from India (45–58%) [25]. None of the previous studies have reported *Shigella* separately. On the other hand a few studies have detected bacterial species like *bacillus*, *klebsiella, proteus* and *pseudomonas species* which we didn’t report. All those variations might be due to differences in ecological abundance of the bacteria, culture techniques used and the time of data collection.

We have, probably for the first time, assessed factors contributing for or preventing from bacterial contamination of vegetables. Tomato was 2.244 times more likely to be contaminated with bacteria while cabbage was 73.5% less likely to be contaminated compared to green pepper. Based on the nature of the vegetables, leaves of cabbage seem to support many bacteria while the smooth surface of tomato less likely holds contaminant bacteria. We call up further study by recruiting equal number of each vegetable in order to drive definitive conclusion. The way vegetables are handled and displayed defers among markets so that the tendency to be contaminated also varies. Vegetables sold in Sikela and Shecha markets were 56.4% and 85.7% less likely to be contaminated by bacteria respectively compared to vegetables sold in Konso sefer.

### Conclusion and recommendation

In conclusion bacterial contamination rate in the present study was significantly considerable. *E. coli* was the commonest bacterial contaminant. Cabbage was the most frequently contaminated vegetable. This alarms the public health sector to work on safe transportation, handling and utilization of contamination prone vegetables as well as continuous screening of on-market vegetables. We also recommend further study by increasing the sample size with equal number of samples from each item considering seasonal variations in order to exhaustively assess contributing factors.

## Limitations

We didn’t measure the intensity (colony count) of contamination due to lack on laboratory facility.
